# Influence of war on quantitative and qualitative changes in drug-induced mortality in Split-Dalmatia County, Croatia

**DOI:** 10.3325/cmj.2011.52.629

**Published:** 2011-10

**Authors:** Ivana Marasović Šušnjara, Marija Definis Gojanović, Davor Vodopija, Vesna Čapkun, Ankica Smoljanović

**Affiliations:** 1 Public Health Institutes of Split and Dalmatian County, Split, Croatia; 2 Department of Pathology and Forensic Medicine, Split University Hospital, Split, Croatia; 3 Croatian Military Academies, Split, Croatia; 4 Department of Nuclear Medicine, Split University Hospital, Split, Croatia

## Abstract

**Aim:**

To study drug-induced mortality and characteristics of overdose deaths in the war (1991-1995), pre-war (1986-1990), and post-war period (1996-2000) in Split-Dalmatia County.

**Methods:**

We retrospectively searched through Databases of the Department of Forensic Medicine, University Hospital Split, the national register of death records, the archives of the Split-Dalmatia County Police, and the Register of Treated Drug Addicts of the Croatian National Institute of Public Health, covering the period from 1986 to 2000, according to drug poisoning codes IX and X of the International Classification of Diseases. The indicators were statistically analyzed.

**Results:**

There were 146 registered drug-induced deaths, with 136 (93%) deceased being men. The median age of all cases was 27 years (interquartile range 8). Most of them were single (70.6%), unemployed (44.6%), and secondary school graduates (69.2%). In the war period, there were 4.8 times more deaths than in the pre-war period (*P* = 0.014), and in the post-war period there were 5.2 times more deaths than in the pre-war period (*P* = 0.008). The most common site of death was the deceased person’s home. The toxicological analyses showed that 59 (61%) deaths were heroin related, alcohol use was found in 62 cases (42.5%), and multi-substance use was found in more than a half of the cases. In 133 (91.1%) cases, deaths were classified as unintentional, whereas 13 (8.9%) were classified as suicides.

**Conclusion:**

The war, along with other risk factors, contributed to unfavorable developments related to drug abuse in Split-Dalmatia County, including the increase in the drug-induced mortality rate.

Drug abuse is among the main causes of health problems and deaths of young people in Europe (1). Drug abuse-related mortality is one of the epidemiological indicators used for the assessment of prevalence and health-consequences of drug abuse (2). When compared with general population of the same age and sex, drug addicts have an increased risk of death (2). This implies both drug-induced deaths and deaths caused by illnesses linked to years-long drug abuse or risk behavior. An important drug-related cause of death is overdose. According to the definition of the European Monitoring Centre for Drugs and Drug Addiction (EMCDDA), drug-induced deaths refer to the deaths caused by the use of one or more substances, at least one of which is a psychoactive drug (3). Although there are differences among countries, the number of drug-induced deaths in the world has significantly increased in the recent few years (2-8).

At the beginning of the War in Croatia 1991-1995, there was an outbreak of addiction epidemics and an increase in the prevalence of drug addiction (9). In the period from 1991 to 1999, the number of drug-addicts increased more than twice. In addition to this, official data on drug-induced deaths for the last few years show a constant increase (10). The increase in the number of drug addicts and fatal drug poisonings since the 1990s has been observed in Split-Dalmatia County as well (11,12).

Several risk factors are identified as related to drug-induced deaths, such as sex, age, comorbid diseases, and pattern and method of drug-taking (taking of more drugs, intravenous use) (13-15).

Since the war has been established as a cause of high and growing rates of morbidity and mortality (16-18), this article examines the changes in the drug-induced mortality rates in the war, pre-war, and post-war period, and analyses the characteristics of drug addicts and the circumstances involved.

## Materials and methods

The sources of data were the archives of the Department of Forensic Medicine, University Hospital Split, the national register of death records, the archives of the Split-Dalmatia County Police, and the Register of Treated Drug Addicts of the Croatian National Institute of Public Health.

Basic and external causes of death were searched according to the EMCDDA definition of drug-induced deaths pursuant to the codes IX (for deaths occurring from 1986 to 1996) and X (for deaths occurring from 1996 to 2000) of the International Classification of Diseases (ICD-lX; ICD-X) (19,20) and they included the following codes: 304 – drug dependence, 305 – drug abuse, E 850.0 – accidental drug poisoning with psychoactive drugs and related narcotics, E854.1 – accidental poisoning with hallucinogens (derivates of cannabis, lysergide (LSD), derivates of marijuana), E854.2 – accidental poisoning with psycho-stimulant substances (amphetamine), E855.2 – accidental poisoning with local analgesics (cocaine), E950.0 – suicide committed with opiates; T40 – poisoning with narcotics and psychodisleptics, X42 – accidental poisoning and exposure to narcotics and psychodisleptics not classified elsewhere, X62 – intentional poisoning and exposure to narcotics and psychodisleptics (hallucinogens) not classified elsewhere, and Y12 – poisoning and exposure to narcotics and psychodisleptics (hallucinogens) not classified elsewhere, undetermined intent. Cases were identified as death due to overdose on the grounds of the coroner’s decision or toxicological analysis results obtained after the autopsies.

The following mortality indicators were used: absolute numbers, shares (%), and specific mortality rates calculated on the basis of 100 000 inhabitants in the age group 15-54 years according to the 1991 Census (period 1986 to 1995) and according to the 2001 Census (period 1996-2000) of the Croatian Bureau of Statistics (21,22). Due to the availability of data on employment of the county inhabitants only for the period from 1990 to 2000, specific rates were calculated only for the war and the post-war period (23,24).

The toxicological analysis results were obtained from the reports on laboratory analysis of blood, urine, and other tissue samples taken during the autopsy (the laboratory of the Institute of Forensic Medicine at the Medical Faculty in Zagreb and the laboratory of the Department of Forensic Medicine, University Hospital Split).

The data were analyzed by Statistica 7.0 (StatSoft^®^ Inc., Tulsa, OK, USA). The statistical analysis included descriptive statistics methods, Pearson χ^2^ test, and Kruskal-Wallis tests. *P* < 0.05 was considered to be statistically significant.

## Results

**Figure 1:**
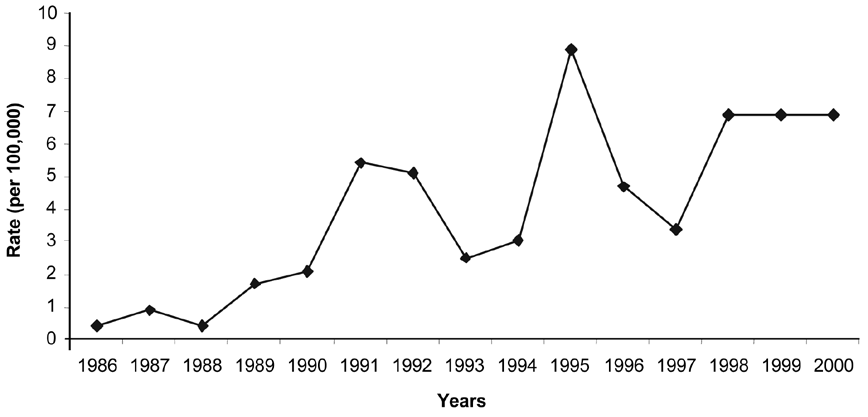
Specific mortality rate of drug-induced deaths in Split-Dalmatia County between 1986 and 2000.

From 1986 to 2000, in Split-Dalmatia County there were 146 registered drug-induced deaths, which indicated an increase in drug abuse-related mortality rate (Figure 1). The mortality rate in the pre-war period was 1.0 (95% confidence interval [CI], 0-2.6), in the war period 4.8 (95% CI, 1.7-7.9), and in the post-war period 5.2 (95% CI, 1.9-8.4).

There were significantly more drug-induced deaths in the war than in the pre-war period (χ^2^_1_ = 6.1, *P* = 0.014), and in the post-war than in the pre-war period (χ^2^_1_ = 7.0, *P* = 0.008).

Most of the deceased were men – 136 (93%), without significant differences between the sub-periods (χ^2^_2_ = 1.9, *P* = 0.379) (Table 1). The median age of all deceased was 27 years (interquartile range 8) (Table 1). Individuals who died were significantly older in the pre- and post-war period than during the war (χ^2^_2_ = 13.1, *P* < 0.001, Kruskal-Wallis test).

**Table 1:** Characteristics of persons who died of overdose in Split Dalmatia County during three time periods between 1986 and 2000

Characteristic	No. (%) of persons who died in				
	prewar (1986-1990)	wartime (1991-1995)	postwar (1996-2000)	total (1986-2000)	*P* (Pearson χ^2^ test)*
Sex:					
male	12 (85.7)	59 (92.2)	65 (95.6)	136 (93.0)	0.379
female	2 (14.3)	5 (7.8)	3 (4.4)	10 (7.0)	
Age in years (median; interquartile range)	26.5;10	25; 8	29;10	27; 8	0.001^†^
Marital status:					
single	9 (64.3)	50 (78.1)	44 (64.7)	103 (70.6)	0.658
divorced/widowed	1 (7.1)	2 (3.1)	2 (2.9)	5 (3.4)	
married	2 (14.3)	5 (7.8)	11(16.2)	18 (12.3)	
unknown	2 (14.3)	7 (10.9)	11(16.2)	20 (13.7)	
Education:					
primary school	3 (21.4)	10 (15.6)	15 (22.1)	28 (19.2)	0.626
secondary school	8 (57.1)	47 (73.4)	46 (67.6)	101 (69.2)	
university	0	1 (1.6)	0	1 (0.7)	
unknown	3 (21.4)	6 (9.4)	7(10.3)	16 (10.9)	
Employment status:					
employed	3 (21.4)	16 (25)	25 (36.8)	44 (30.1)	0.006
unemployed	3 (21.4)	28 (43.8)	34 (50.0)	65 (44.6)	
student	4 (28.6)	7 (10.9)	2 (2.9)	13 (8.9)	
unknown	4 (28.6)	13 (20.3)	7 (10.3)	24 (16.4)	
Place of death:					
residence	7 (50.0)	19 (29.7)	29 (42.6)	55 (37.7)	0.361
other inside	2 (14.3)	14 (21.9)	17 (25.0)	33 (22.6)	
outside	2 (14.3)	23 (35.9)	12 (17.7)	37 (25.3)	
unknown	3 (21.4)	8 (12.5)	10 (14.7)	21 (14.4)	
Area of death:					
Split	11(78.6)	54 (84.4)	43 (63.2)	108 (74.0)	0.048
rest of the county	3 (21.4)	10 (15.6)	25 (36.8)	38 (26.0)	
Day of death:					
Monday	3 (21.4)	3 (4.7)	8 (11.8)	14 (9.6)	0.073
Tuesday	2 (14.3)	6 (9.4)	8 (11.8)	16 (10.9)	
Wednesday	0	8 (12.5)	7 (10.3)	15 (10.3)	
Thursday	4 (28.6)	14 (21.9)	12 (17.6)	30 (20.4)	
Friday	2 (14.3)	11 (17.2)	9 (13.2)	22 (15.1)	
Saturday	2 (14.3)	12 (18.8)	14 (20.6)	28 (19.1)	
Sunday	1 (7.1)	10 (15.6)	10 (14.7)	21 (14.3)	
Crime:					
no	9 (64.3)	37(57.8)	20(29.4)	66 (45.2)	0.001
yes	5 (35.7)	27(42.2)	48 (70.6)	80 (54.8)	
Toxicological findings					
negative	1 (7.1)	20 (31.3)	11 (16.7)	32 (21.9)	0.001
positive	1 (7.1)	17 (26.6)	47 (71.2)	65 (44.5)	
unknown/not done	12 (85.7)	27 (42.1)	10 (12.1)	49 (33.6)	
Alcohol detected:					
no	0	8 (12.5)	25 (37.9)	33 (22.6)	0.039
yes	3 (21.4)	28 (43.8)	31 (47.0)	62 (42.5)	
unknown/not done	11 (78.6)	28 (43.8)	12 (15.1)	51 (34.9)	
Total	14 (100)	64 (100)	68 (100)	146 (100)	

*Statistical difference between examined periods (*P* < 0.05 is significant).

†Kruskal-Wallis test.

Most of the deceased were single – 103 (70.6%) during the entire period, and there was no significant differences between the sub-periods (χ^2^_6_ = 4.1, *P* = 0.658) (Table 1).

According to the level of education, most of the deceased – 101 (69.2) – had completed secondary school education, whereas only one (0.7%) had college/university education in the entire period. Also, there was no significant difference between the sub-periods (χ^2^_6_ = 4.4, *P* = 0.626) (Table 1). Most of deceased were unemployed – 65 (44.6%) in the entire period, with a significant difference between the sub-periods (χ^2^_6_ = 21.6, *P* = 0.006) (Table 1).

Specific mortality rate for the employed was 2.8 (95% CI, 0-7) in the war period and 5.4 (95% CI, 0-11.1) in the post-war period; mortality rate for the unemployed was 15.4 (95% CI, 0-30) in the war period and 14 (95% CI, 0-27.5) in the post-war period. There were significantly more unemployed than employed among the deceased (χ^2^_1_ = 7.2; *P* = 0.007). In the post-war period, the probability of a fatal outcome was 90% higher among the unemployed than among the employed (χ^2^_1_ = 2.6; *P* = 0.102).

The greatest number of the deceased in relation to the total number of inhabitants was in Split, the capital of the county, both in the entire period and in the individual sub-periods (χ^2^_2_ = 12.7, *P* = 0.048) (Table 1).

A total of 55 (37.7%) deaths occurred in the deceased persons’ residence and 8 (12.0%) in someone else’s residence, with no significant difference between the sub-periods (χ^2^_6_ = 15.2, *P* = 0.361) (Table 1). Actually, most of the deaths took place indoors. There were 21 (14.4%) deaths registered outdoors, in public spaces. One-third of the deaths occurred on Saturday or Sunday, with no significant difference between weekend and work days (χ^2^_6_ = 14.5, *P* = 0.073) (Table 1). Slightly more than 50.0% of the deceased had committed a criminal offense during their lifetime, with significant difference between the sub-periods (χ^2^_2_ = 13.0, *P* < 0.001) (Table 1). In the entire period from 1986 to 2000, autopsies were performed if requested by an investigative judge or coroner. A total of 127 (86.9%) of the deceased were autopsied. A toxicological analysis was performed in 97 (44.5%) deceased, with a significant difference between the sub-periods (χ^2^_6_ = 50.0, *P* < 0.001) (Table 1).

Death was heroin-related in 59 (61.0%) cases for which the toxicological analysis was performed. The second most frequent positive substance, in 62 (42.5%) cases, was alcohol. In 17 (17.5%) cases, consumption of benzodiazepines was confirmed. Fifty one deceased persons (52.6%) were under simultaneous influence of more than one substance at the moment of death. A hundred and thirty-three deaths (91.1%) were unintentional and 13 (8.9%) were suicides.

## Discussion

In the period from 1986 to 2000, there was a significant increase in the absolute number and mortality rate of drug-induced deaths in Split-Dalmatia County. The increase was most heavily pronounced in the war period, when the rates increased by almost five times in comparison with the pre-war period. The constant growth was continued in the post-war period as well.

Following the war, Croatia was faced with a set of social and economic changes (25,26). Moral crisis and erosion of the value system, difficult economic situation, inadequate functioning of the rule of law, all contributed to the increase in drug supply and demand (9,27,28).

Most fatal cases in entire period were men, without differences between war, pre-war, and post-war sub-periods, which reflects a higher probability of addiction development in men than in women (29,30). However, when the number of people who are really threatened by drug overdose is monitored, the men-women ratio is reduced to 1:5, which suggests that female drug users are exposed to a similar risk as male (29,31). Individuals who died of drug abuse were averagely older in the pre- and post-war sub-period than during the war, while in the entire period they were on average in their late twenties and early thirties, which corresponds to reports in the literature (4,5,8,29,30). This also confirms that, contrary to the popular belief, young, inexperienced drug users are not in the greatest danger of drug overdose (13,14). Especially at risk are those who were rarely enrolled in medical treatment (13). Unfortunately, we did not collect the data on whether the deceased persons were enrolled in medical treatment at the moment of their death.

This research showed that most of the deceased had been involved in some criminal activities and underwent court proceedings in the course of their lives, especially in the post-war period. Numerous studies consistently indicate a significantly higher rate of drug addicts than members of the general population among offenders (32-34). Also, many studies reported that prisoners’ chief motive for committing a criminal offense was drug procurement (32,33). If we add the fact that most of the deceased were unemployed, then the data referring to their criminal past are even more understandable. Furthermore, unemployment indirectly increases the risk of drug-induced mortality regardless of other factors (18,35), which was also confirmed by this research. The fatal outcomes among the employed could be explained by their being unsatisfied, frustrated, or hedonism-inclined.

As regards the place of incident, this research found that the deaths took place in enclosed spaces, such as the victims’ or someone else’s residences, hotel rooms, toilettes or in public spaces such as parks and streets, which was not changed during the entire study period. This is in line with other studies, although some studies found that most of the deaths occurred in public spaces, usually in the streets (3,5,8).

The majority of deaths took place in Split, the capital of the County, significantly more in war and post-war than in the pre-war period. Other studies also showed that some causes of death, including drug abuse, were more prevalent in urban areas (36). Most probably it is an immediate consequence of higher prevalence of addicts, as well as of greater availability of drugs (35,36).

This study did not find more drug-induced deaths on weekend days, unlike the study by Darke et al (37). Such finding, however, is in line with other studies on drug-induced deaths (3,38), which focused on the unemployed population using drugs on a regular basis, and not only recreationally on weekends. Most of the deceased had been single during their lifetime, which did not change during the observed period. Demographic studies show that single people are exposed to a significantly higher drug abuse risk and have a higher all-cause mortality risk (39,40). Being married probably has an opposite influence (35). The greatest number of the deceased had secondary school education, although a large number also had only primary school. It has been proved that higher education is associated with less drug abuse (35) and that the level of education significantly correlates with all mortality causes (29,33). This research confirms the low socio-economic status and a low level of education in the deceased, similar to some other studies (35,41).

Heroin (or its metabolites) was the most frequently identified drug – alone or in a combination with some other drugs and alcohol. These drugs were also the cause of the most drug-related deaths in Europe (4). A positive toxicological result for several substances in most of the drug-related deaths is in line with studies in other countries that show the connection between the consumption of several substances and overdose deaths (42,43). Use of other substances (benzodiazepines and analgesics) and alcohol is mostly mentioned as an additional risk factor (8,30,44,45). Several studies have established that even a small quantity of alcohol in combination with heroin represents a risk factor (46,47).

The negative results of toxicological analysis found in some cases can partly be explained by the instability of the heroin molecule. Besides, possible inadequate sampling, poor state of the body, and redistribution of metabolites may have limited the interpretation of analytical results. However, fresh puncture wounds and/or syringes with narcotic drugs found in the vicinity of the deceased indicated that the drug may have been injected.

Most of the deaths were found to be accidental and about 10% were suicides. Although in most cases overdoses occur accidentally, it is established that a suicide rate among heroin users is 14 times higher than in the general population (42). Recent cohort studies in Europe show that between 6% and 11% of deaths among drug users are suicides. However, the total prevalence of these causes is very difficult to estimate due to the limited information (4). According to the mentioned findings, there are some assumptions that many overdoses were not classified as suicide. However, some studies found the opposite. For instance in Australia, only 5% of drug-induced deaths were classified as suicide, and almost all heroin users claimed that their “non-fatal” overdoses were accidental and not intentional (14,30,37).

This research used different sources of data because routine mortality statistics show only demographic data and the cause of death, tending to underreport the number of overdose deaths and not reflecting a more specific cause of deaths (48).

To sum up, our data confirmed a significant increase in the drug-induced mortality rate in Croatia during the last two decades. The increase that began during the war persisted through the post-war period and is observed not only in our country but all over the world (2-9,12,29,45,49-54). Our data firmly support the already proven death-related risk factors such as sex, age, unemployment, criminal behavior, and use of other drugs in combination with heroin. The war and the post-war situation, together with other risk factors, certainly contributed to stirring up of the problems related to drug abuse in Split-Dalmatia County, increasing the number of addicts and drug-induced mortality rate.
